# Cross-Species Metabolomic Analyses in the Brassicaceae Reveals Common Responses to Ultraviolet-B Exposure

**DOI:** 10.1093/pcp/pcad085

**Published:** 2023-08-12

**Authors:** Yue Jing, Mutsumi Watanabe, Fayezeh Aarabi, Alisdair R Fernie, Monica Borghi, Takayuki Tohge

**Affiliations:** Max-Planck Institute for Molecular Plant Physiology, Central Metabolism, Am Mühlenberg 1, Potsdam-Golm D-14476, Germany; Division of Biological Science, Nara Institute of Science and Technology (NAIST), Ikoma, 630-0192 Japan; Max-Planck Institute for Molecular Plant Physiology, Central Metabolism, Am Mühlenberg 1, Potsdam-Golm D-14476, Germany; Max-Planck Institute for Molecular Plant Physiology, Central Metabolism, Am Mühlenberg 1, Potsdam-Golm D-14476, Germany; Department of Biology, Utah State University, 5305 Old Main Hill, Logan, UT 84321-5305, USA; Max-Planck Institute for Molecular Plant Physiology, Central Metabolism, Am Mühlenberg 1, Potsdam-Golm D-14476, Germany; Division of Biological Science, Nara Institute of Science and Technology (NAIST), Ikoma, 630-0192 Japan

**Keywords:** Brassicaceae, Phenylacylated flavonoid glycosides, Saiginols, Sinapoyl derivatives, UV-B stress

## Abstract

Exposure to UV-B radiation, an intrinsic component of solar light, is detrimental to all living organisms as chromophore units of DNA, RNA and proteins readily absorb high-energy photons. Indirect damage to the same molecules and lipids is mediated by elevated reactive oxygen species (ROS) levels, a side effect of exposure to UV-B stress. To protect themselves from UV-B radiation, plants produce phytochemical sunscreens, among which flavonoids have shown to be particularly effective. The core aglycone of flavonoid molecules is subjected to chemical decoration, such as glycosylation and acylation, further improving sunscreen properties. In particular, acylation, which adds a phenolic ring to flavonoid molecules, enhances the spectral absorption of UV-A and UV-B rays, providing to this class of compounds exceptional shielding power. In this study, we comprehensively analyzed the responses to UV-B radiation in four Brassicaceae species, including *Arabidopsis thaliana, Brassica napus, Brassica oleracea,* and *Brassica rapa.* Our study revealed a complete reprogramming of the central metabolic pathway in response to UV-B radiation characterized by increased production of functional precursors of specialized metabolites with UV-B shielding properties, indicating a targeted effort of plant metabolism to provide increased protection. The analysis of specialized metabolites and transcripts revealed the activation of the phenylpropanoid–acetate pathway, leading to the production of specific classes of flavonoids and a cross-species increase in phenylacylated-flavonoid glucosides with synapoyl glycoside decorations. Interestingly, our analysis also revealed that acyltransferase genes of the class of serine carboxypeptidase-like (SCPLs) proteins are costitutively expressed, but downregulated in response to UV-B radiation, possibly independently of the ELONGATED HYPOCOTYL 5 (HY5) signaling pathway.

## Introduction

Exposure to high-frequency wavelengths of the solar ultraviolet-B radiation (280–320 nm) has the potential to cause structural modification to DNA, RNA, and proteins as UV-B chromophores present in these molecules readily absorb high-energy photons associated with short-wavelength radiation. Additionally, indirect damage to the same macromolecules and also lipids is caused by the concomitant production of reactive oxygen species (ROS), as these small chemicals containing oxygen become very reactive after accepting high-energy electrons (Halliwell and Gutteridge 1985, Jenkins 2009). Therefore, photoautotrophic organisms with obligate dependence on sunlight exposure for the synthesis of carbohydrates have evolved strategies to cope with both the direct and indirect effects of UV-B. At low concentrations, endogenously produced ROS act as cellular signals for cell growth and development (Muhlemann et al. 2018, Chapman et al. 2019). Thus, enzymatic scavengers mainly contribute to ROS quenching and maintenance of ROS cellular homeostasis under standard growth conditions (Waszczak et al. 2018). Conversely, plant-specialized metabolites protect against both direct and indirect UV-B damage associated with either transitory elevated UV-B exposure, for example, in days of high solar radiance, or steady elevated solar radiance typical of low-to-middle latitude and high-altitude zones (Tohge et al. 2016, Chapman et al. 2019). Typically, UV-B protective molecules are constitutively expressed in plants adapted to habitats with high solar irradiation. Nonetheless, specific responses leading to novel molecule synthesis can also be induced upon UV-B exposure, following the activation of the UV RESISTANCE LOCUS 8 (UVR8) UV-B receptor pathway (Rizzini et al. 2011). UVR8 monomers, deriving from the dissociation of UVR8 homodimers after the absorption of high-energy photons, free proteins from proteasomal degradation by interacting with the CONSTITUTIVELY PHOTOMORPHOGENIC 1 (COP1) E3 ubiquitin ligase and concurrently activate the UV-B signaling cascade (Yin et al. 2015, Lau et al. 2019). The basic leucine zipper transcription factor ELONGATED HYPOCOTYL 5 (HY5) is a leading downstream regulator of the UVR8–COP1 response, which, in the dark, is perpetually degraded via COP1 ubiquitin activation (Podolec et al. 2021). HY5 induces the transcriptional regulation of more than 3,000 genes, including genes of the core of the phenylpropanoid pathway as well as enzymes contributing to the decoration of flavonoids produced along the phenylpropanoid–acetate branch (Ulm et al. 2004, Stracke et al. 2010, Rai et al. 2019, Shi and Liu 2021, Job et al. 2022). Plants produce several classes of UV-B protective molecules, and flavonoids constitute one of the most potent groups among all phytochemical sunscreens (Tohge and Fernie 2017). The backbone of a typical flavonoid molecule, which is made of two benzene rings linked by a heterocyclic pyran ring (C6-C3-C6), can absorb high-energy photons and also donate electrons to unstable ROS molecules (Chapman et al. 2019). Hence, flavonoids are structurally built to protect against the direct and indirect effects of UV-B radiation, and in fact, the phenylpropanoid–acetate pathway evolved early during the evolutionary history of the green lineage when plants left water and conquered land (Rensing 2018). Flavonoids are grouped into six major classes based on their aglycone moiety, which can be further modified by chemical decorations such as glycosylation and acylation (Tohge et al. 2018). The addition of sugar molecules increases the solubility of the aglycone, for which glycosylated flavonoids can accumulate in higher concentrations inside the vacuoles of epidermal cells. In so doing, they form a superficial layer of phytochemical sunscreen that shields UV-B rays from penetrating deeper inside the tissues (Stracke et al. 2010). Additional decorations, such as the presence of acyl groups, can further increase the UV-B-absorbing properties of flavonoids as demonstrated for the phenylacylated-flavonoid glycoside saiginol A, identified in an LC-MS screen of *Arabidopsis thaliana* flowers (Tohge et al. 2016). The presence of the additional phenolic ring deriving from the sinapoyl decoration enhances the spectral absorption of UV-A and UV-B rays, providing to saiginols exceptional sunscreen power. The acylation of saiginol A is catalyzed by the flavonol-phenylacyltransferase 2 (FPT2) initially annotated as a serine carboxypeptidase-like (SCPL) protein. In *A. thaliana*, the *FPT2* gene is located on chromosome 2 in a cluster with seven *SCPL* genes. However, only saiginol-producing accessions with geographical distribution in areas with elevated UV-B radiance carry a functional *FPT2* gene, indicating that individual mutations have possibly been filtered out by natural selection in areas with a low solar radiance. While orthologs of the *FPT2* gene were only found in *Arabidopsis lyrata*, cross-species analyses identified two syntenic blocks of *SCPL* genes in *Brassica rapa*.

Here, by building on previous discoveries, we expanded the research on phenylacylated-flavonoid glycosides to *Brassica napus*, *B*. *oleracea*, and *B. rapa*. Our analyses included a comprehensive annotation of primary and specialized metabolites and expression patterns of *HY5* and *SCPL* genes in response to UV-B radiation.

## Results and Discussion

### Experimental design

*Arabidopsis thaliana* plants respond very rapidly to UV-B irradiation. After just a few seconds of exposure to UV-B light, UVR8 homodimers dissociate into monomers, initiating the UV-B signaling cascade (Rizzini et al. 2011). While transcriptional activation is very fast, metabolic responses require longer times to be deployed as they depend on cycles of novel metabolite synthesis and degradation (Kusano et al. 2011). Moreover, the progressive accumulation of metabolites in specific intracellular organelles also requires time, as the process often encompasses the chemical decoration of metabolites occurring in the cytosol, followed by the vesicular transport of compounds into the vacuole (Chanoca et al. 2015). Taking these considerations into account, in this study, we investigated time-point transcriptional and metabolic responses to UV-B light exposure in a comparative experiment between *A. thaliana* ecotype Col-0 and three species of the Brassicaceae family, namely *B. napus, B. oleracea* and *B. rapa*. More specifically, we assessed the responsiveness of each species to promptly activate the transcriptional response to UV-B radiation, which, during the early stages of plant life, is essential for the progression of vegetative development. For this, we assessed the transcriptional responses of genes known to be induced by UV-B exposure in 2-week-old seedlings at seven consecutive time points (0.25, 0.5, 1, 2, 6, 12 and 24 h). Still, whether adult plant can maintain a balanced metabolic state is also relevant for transitioning into the reproductive stage. Therefore, we further assessed changes in primary and specialized metabolite abundance in 4-week-old plants exposed to UV-B light for 2 and 10 h, as well as the abundance of transcripts of key genes in the pathway of flavonoid synthesis at 6 and 8 h. Plant samples harvested immediately before the beginning of the UV-B treatment (time point 0 h) were used as negative controls for all measurements. At the beginning of the UV-B treatment, all seedlings and adult plants have been exposed to 3 h of visible photosynthetic light devoid of UV-B frequencies; hence, they were actively photosynthesizing.

### Transcriptional responses to UV-B exposure in young seedlings and expression of *SCPL* genes

**Fig. 1:**
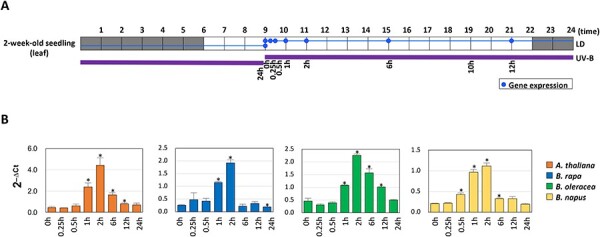
The transcriptional activation of *HY5* in response to UV-B exposure. (A) A schematic representation of the duration of the UV-B treatment (thick purple line) and time points of sample harvest (dots on the thin blue line). Gray and white squares represent dark and light photoperiods, respectively. (B) The abundance of *HY5* transcripts normalized by the expression of *UBQ10* at different time points during the UV-B treatment. Each bar represents the means ±  Standard Error (SE) (*n* = 3). Bars marked with an asterisk are significantly different at *P* < 0.05. LD, length of the day.

In *A. thaliana*, the response to UV-B light is mostly regulated at the level of transcription, with HY5 acting as a leading regulator in the process (Podolec et al. 2021). After UVR8–COP1-mediated posttranslational stabilization, HY5 protein begins accumulating in the nucleus, where it binds to the regulatory region of numerous genes and activates the UV-B response. Transcriptional activation of genes directly involved in flavonoid synthesis such as *MYB12, CHALCONE SYNTHASE (CHS),* and *CHALCONE ISOMERASE (CHI)* was also shown to be mediated by HY5 acting together with zinc-fingers of the BBX family, which provide the transcriptional activation potential (Job et al. 2022, Podolec et al. 2022). The results of a recent analysis of transcripts and genomic sequences suggested that the core UVR8–COP1–HY5 signaling pathway originated in a common ancestor when plants emerged from water and developed common means of protection toward the damaging effect of the solar radiation (Han et al. 2019). To investigate whether the activation response by HY5 is conserved across the Brassicaceae species, we measured the steady-state level of HY5 transcript abundance in 2-week-old seedlings at specific time points of exposure to UV-B radiation (**Fig. 1A**). In all species, including *A. thaliana, HY5* expression peaks 2 h after exposure to UV-B light, which indicates that the activation of the response to UV-B radiation proceeds in all Brassicaceae species with a similar trend. Moreover, despite the accumulation of *HY5* transcripts starts to decrease after 2 h of UV-B treatment, it is still significantly elevated after 6 h of exposure or longer (**Fig. 1B**). Moreover, significant levels of *HY5* transcripts are already evident after 1 h of exposure or just 30 min as in *B. napus*. These results revealed that the pattern of *HY5* expression is well conserved across all Brassicaceae species and characterized by a strong and prompt initial induction happening soon after the first exposure to UV-B irradiation.

**Fig. 2:**
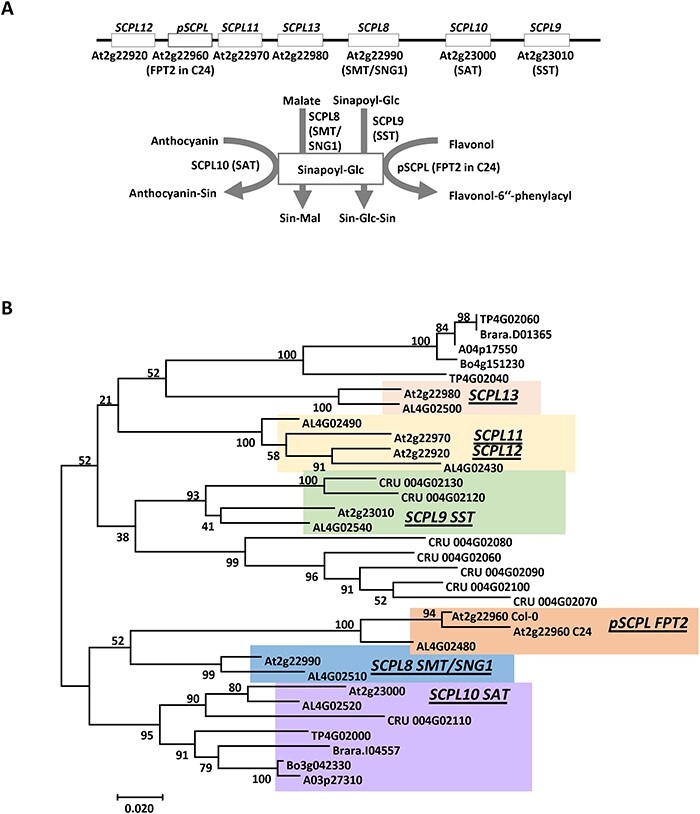
The *A. thaliana SCPL* gene cluster and phylogenetic analysis of *SCPL* genes in the Brassicaceae. (A) A schematic representation of the *SCPL* gene cluster on chromosome 2 of *A. thaliana* and the metabolic function of characterized genes in the cluster. (B) The phylogenetic tree of *SCPL* genes in Brassicaceae. SNG1, sinapoyl-glucose accumulator 1.

**Fig. 3:**
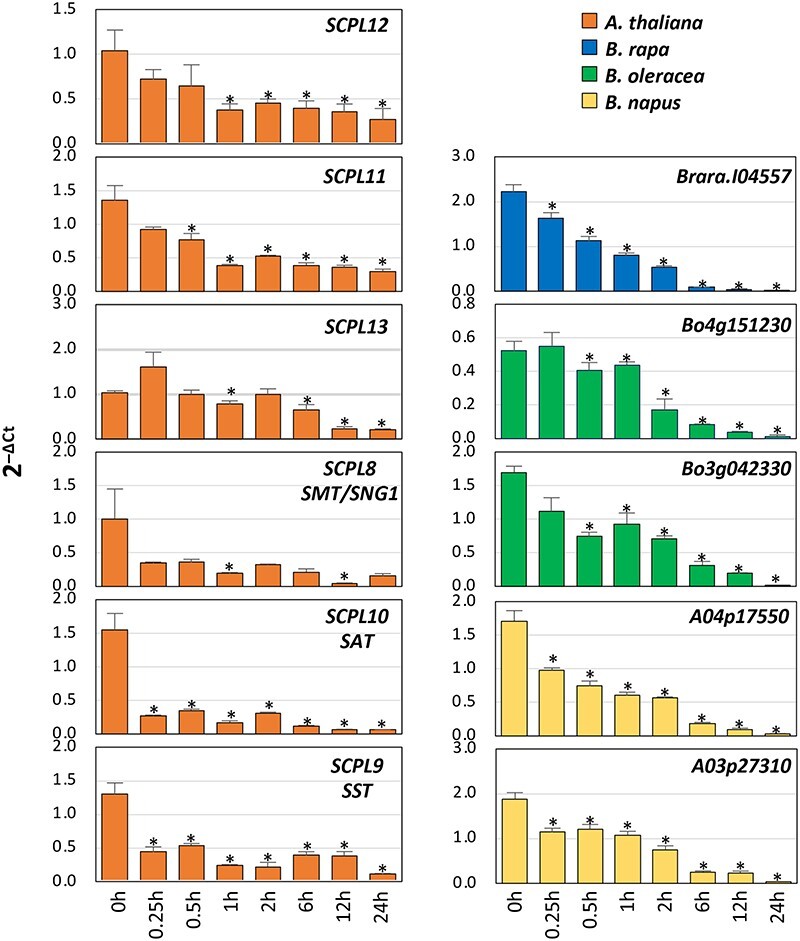
The abundance of *SCPL* transcripts in response to UV-B exposure. The abundance of *SCPL* transcripts normalized by the expression of *UBQ10* at different time points during the UV-B treatment. Each bar represents the means ±  Standard Error (SE) (*n* = 3). Bars marked with an asterisk are significantly different at *P* < 0.05.

In *A. thaliana*, a member of the SCPL family named FPT2 was shown to catalyze the acylation of the recently characterized phenylacylated-flavonoid glycoside saiginol A, which confers increased fitness to ecotypes adapted to areas with high solar radiance (Tohge et al. 2016). The *FPT2* gene localizes on chromosome 2 in a tandem duplication with seven *SCPL* genes (**Fig. 2A**). The tandem duplicated gene cluster includes *SCPL12* (*FPT1*, At2g22920), *pSCPL* (*FTP2*, At2g22960), *SCPL11*, (At2g22970), *SCPL13* (At2g22980), *SCPL8* [sinapoyl-glucose:malate sinapoyltransferase (SMT), At2g22990], *SCPL10* [sinapoyl-glucose: anthocyanin sinapoyltransferase (SAT), At2g23000], and *SCPL9* [sinapoyl-glucose:sinapoyl-glucose sinapoyltransferase (SST), At2g23010]. The seven *SCPL* genes display high sequence similarity and share a common sinapoyl donor but have specific sinapoyl acceptors. In this study, orthologs of *A. thaliana SCPL* genes in the Brassicaceae species were identified by comparative genomic analysis using syntenic relationships and utilized to build a phylogenic tree in which the known *A. thaliana SCPL* genes encoding for SAT, FPT2, SMT and SST enzymes served for the identification of potential functional clades (**Fig. 2B**). As genes belonging to a same clade are supposed to have a similar enzymatic function, with this method, we identified the putative *SCPL* genes A04p17550 and A03p27310 in *B. napus*, Bo4g151230 and Bo3g042330 in *B. oleracea,* and Brara.I04557 in *B. rapa*. Therefore, on the same set of samples described earlier, in which overexpression of *HY5* displayed UV-B-dependent induction, we measured the level of transcripts of *SCPL* genes and the orthologs. Conversely to our expectations, the results of these experiments showed that all *SCPL* genes, irrespective of the species, displayed a progressive downregulation upon UV-B exposure, revealing that *SCPL* expression is constitutive rather than inducible (**Fig. 3**). Whether HY5 is responsible for the downregulation of *SCPL* genes in response to UV-B is intriguing to postulate. However, this hypothesis would be better resolved in *hy5* mutant lines of all the Brassicaceae species. Nonetheless, as the pattern of *SCPL* expression does not follow the pattern of *HY5* transcriptional activation, it is possible that the *SCPL* expression is governed by a different transcriptional activator.

### Metabolites of the central pathway displayed the accumulation of functional precursors for targeted specialized metabolite synthesis across all Brassicaceae species

**Fig. 4:**
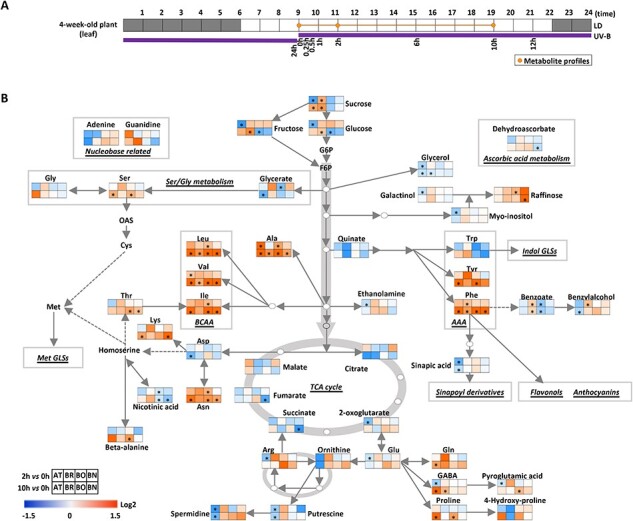
Metabolites of the central pathway. (A) A schematic representation of the duration of the UV-B treatment (thick purple line) and time points of sample harvest (dots on the orange line). Gray and white squares represent dark and light photoperiods, respectively. (B) A schematic representation of the central metabolic pathway. Heatmaps along the pathway represent log_2_ fold changes of metabolite abundance measured in leaves at time point 2 and 10 h versus time 0. Metabolites marked with an asterisk are significantly different at *P* < 0.05. AAA, aromatic amino acid; AT, *A. thaliana*; BCAA, branched amino acids; BN, *B. napus; BO*, *B. oleracea*; BR, *B. rapa*; F6P, fructose-6-phosphate; GABA, γ-aminobutyric acid; GLS, glucosinolate; G6P, glucose-6-phosphate; OAS, *O*-acetyl-serine; Sin, sinapoyl; LD, long-day; TCA, tricarboxylic acid cycle.

As the metabolites of the central pathway provide precursors for the synthesis of specialized metabolites, we utilized a well-established gas chromatography–mass spectrometry method (Lisec et al. 2006, Alseekh et al. 2021) to investigate the effect of UV-B radiance on the steady-state accumulation of these compounds. Then, to facilitate data interpretation, peak areas of each specific metabolite measured after 2 and 10 h of exposure were compared to time 0 and plotted on a heatmap of metabolite abundance distributed along the graphical representation of the central pathway (**Fig. 4**; **Supplementary Dataset S1**). As it has already been shown that UV-B radiation induces significant metabolic changes in young seedlings (Kusano et al. 2011), we focused our research on adult plants, in which the maintenance of a balanced metabolic state is relevant for the maintenance of plant physiological functions and transitioning into the reproductive stage. Starting from the pathway of glycolysis, our measurements revealed species-specific responses to UV-B exposure as metabolites and carbohydrates immediately related to the pathway differentially accumulated in the four species under study. Indeed, while the content of the three major sugars (glucose, fructose, and sucrose) significantly decreased in Col-0 after only 2 h of exposure, glucose and fructose slightly increased in all the remaining Brassicaceae, with a significant increment for sucrose in *B. rapa.* After 10 h of treatment, the responses of *B. rapa* and *A. thaliana* started to align, while *B. napus* and *B. oleracea* were still clustered together. The metabolic responses were more leveled across species and time points for the sugar alcohols glycerol, galactinol, and myoinositol. Nevertheless, *A. thaliana* displayed a significant reduction in the content of all these metabolites. High raffinose was measured in *B. napus* and partially in *B. oleracea*, which may reflect a particular form of carbon mobilization typical of these species. In general, the responses observed for Col-0 align with the relative metabolite abundance recently measured after 24 and 96 h of UV-B treatment, which indicates that the decline in sugar content progressively aggravates over long-time exposure (Job et al. 2022).

The pressure exerted by UV-B light on the intermediates of the tricarboxylic acid (TCA) cycle was also elevated in Col-0, as citrate, 2-oxoglutarate, succinate, and fumarate displayed reduced contents. Cross-species differences leveled off for the content of many amino acids, including the branched amino acids Leu, Val, and Ile, as well as Ser, Lys, Thr, and Asn, which all displayed significant increases over 10 h of UV-B exposure. A similar trend was observed for the aromatic amino acids, of which the content progressively increased over time. Interestingly, despite UV-B exerting differential pressure on specific metabolite nodes based on the species, Phe, the precursor of phenylpropanoid metabolism, uniformly increased in all species. A similar trend was observed for the metabolites of two branches of the Met biosynthetic pathway, which starts from Ser and Asp, and diverts toward Thr and Ile on one branch and Cys and Met on the other. As the biosynthesis of phenolic compounds requires significant methylation, it is compelling to speculate that given the high demand for the cofactor *S*‐adenosylmethionine for methylation reactions, all species equally invest in providing a sufficient number of precursor molecules to face increased methylation demand. Pro, the metabolic marker of stress responses in most plants (Ghosh et al. 2022), only increased significantly in Col-0 and *B. oleracea*. Finally, it is worth mentioning the slight increase in dehydroascorbate observed in all species except for *B. napus*. Cellular ROS production is a universal response in organisms exposed to UV-B rays and handled, in the first place, by enzymatic scavengers. Ascorbate peroxidases utilize ascorbate as a reducing agent to detoxify hydrogen peroxide, and in doing so, they release monodehydroascorbate, which spontaneously converts into dehydroascorbate. As the intracellular pool of glutathione is gradually consumed when exposure to UV-B is protracted, less glutathione becomes available to the dehydroascorbate reductase enzyme, for which dehydroascorbate may accumulate in plant tissues.

In conclusion, UV-B light significantly impacted the central metabolic pathway as inferred from changes in metabolite content observed after 2 and 10 h of UV-B exposure. Nonetheless, all Brassicaceae displayed increased production of functional precursors of specialized metabolites with UV-B shielding properties, indicating a targeted effort of plant metabolism to provide increased protection.

### Analysis of specialized metabolites and transcripts revealed species-specific production of UV-B-protective compounds and conserved production of sinapoyl glucose

The transcriptional activation of the flavonoid pathway in response to UV-B light has been reported in many species, which highlights the ancestral origin of this response now shared among plants adapted to life on land (Han et al. 2019). Studies mostly performed in knockout mutants of *A. thaliana* revealed the vital role of flavonoids for plant fitness under UV-B light (Ruegger and Chapple 2001, Dean et al. 2014), while screens of natural accessions identified classes of compounds with enhanced sunscreen shielding properties, which are typical of ecotypes adapted to environments with strong solar radiance (Tohge et al. 2016). Therefore, we assessed whether adult plants are also capable of mounting a transcriptional response to UV-B radiation, which ultimately will induce the activation of the pathway of flavonoid synthesis. For this, we harvested the aboveground portion of plants exposed to UV-B light and extracted RNA and metabolites for further analysis.

**Fig. 5:**
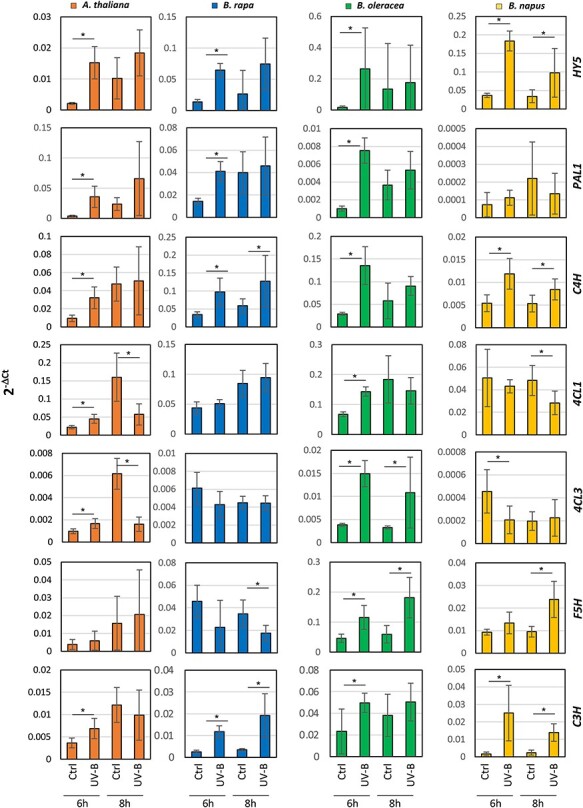
The transcriptional activation of the phenylpropanoid pathway in response to UV-B exposure. Transcript abundance normalized by the expression of *UBQ10* after 6 and 8 h of exposure to UV-B light. Each bar represents the means ±  Standard Error (SE) (*n* = 6). Bars marked with an asterisk are significantly different from the untreated control at *P* < 0.05.

First, we measured the expression of *HY5* and observed that, in all species, the number of transcripts was significantly elevated till 6 h upon UV-B exposure and up to 8 h in *B. napus* (**Fig. 5**). Then, we measured the level of transcripts of key genes of the core phenylpropanoid pathway, as well as genes located after the branching point between the phenylpropanoid and the phenylpropanoid–acetate pathways, such as *FERULIC ACID 5-HYDROXYLASE* (*F5H*) and *COUMARATE 3-HYDROXYLASE* (*C3H*). Upon 6-h exposure to UV-B light, a general activation of the core phenylpropanoid pathway was revealed by the accumulation of transcripts of *PHENYLALANINE AMMONIA-LYASE* (*PAL*), *CINNAMATE 4‐HYDROXYLASE* (*C4H*), and *4‐COUMARATE:CoA LIGASE* (*4CL1* and *4CL3*) genes. These results align with what we had inferred from the analysis of primary metabolites, where we measured an increased content of Phe, which uniformly appeared in all species under study (**Fig. 4**). Similarly, transcripts of *F5H* and *C3H* genes, which are involved in the biosynthesis of lignin (Alber et al. 2019, Sakamoto et al. 2020), revealed a general increase upon UV-B exposure.

**Fig. 6:**
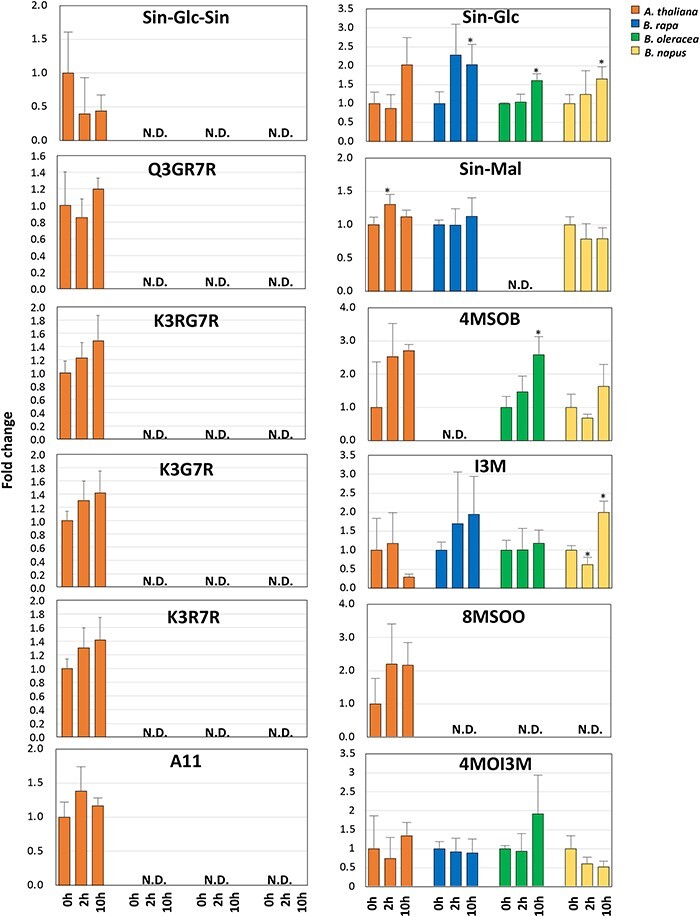
Specialized metabolites. The abundance of the major classes of metabolites measured in leaves of *A. thaliana* ecotype Col-0, *B. napus, B. oleracea* and *B. rapa* exposed to UV-B radiation for 2 and 10 h. Data are represented as fold change of metabolite abundance versus time 0 h. Metabolites marked with an asterisk are significantly different at *P* < 0.05. G, glocoside; K, kaempferol; Mal, malate; Q, quercetin; R, rhamnoside; Sin, sinapoyl; 4MSOB, 4-methylsulfinylbutyl glucosinolate; 8MSOO, 8-methylsulfinyloctyl glucosinolate; N.D., non-detected.

As the results of these measurements revealed a consistent transcriptional activation of the phenylpropanoid pathway, to assess whether transcriptional responses are followed by the increased accumulation of specialized metabolites, we applied a liquid chromatography–mass spectrometry method (Tohge and Fernie 2010, Perez de Souza et al. 2021) to separate and annotate compounds and determine their accumulation at a very early (2 h) and late (10 h) time points upon exposure to UV-B light. Our analysis identified a total of 34 compounds in *A. thaliana* ecotype Col-0, 49 compounds in *B. napus*, 45 in *B. oleracea,* and 52 in *B. rapa*, with 18 compounds shared among all species (**Fig. 6**; **Fig. S1**; **Supplementary Dataset S2**). Thirteen compounds were previously identified by co-elution peaks of leaf extracts from *A. thaliana* (Tohge et al. 2016), while additional 11 compounds were annotated by the interpretation of specific in-source fragmentation patterns of the aglycone moiety and the corresponding decorated compound. Finally, to facilitate cross-species comparisons, peak areas measured for each compound after 2 and 10 h of UV-B treatment were compared to time 0, plotted on a heatmap of clustered metabolites based on similar patterns of abundance (**Fig. S1**), and the metabolites revealing the most significant changes were represented as bar plots (**Fig. 6**). Overall, our analysis revealed a temporal decrease in indolic glucosinolate compounds contrasted by an increase in flavonoids including anthocyanin derivatives and aliphatic glucosinolates in *A. thaliana*. This pattern of metabolite production aligns well with the results reported earlier by Kusano et al. (2011), who measured the metabolite content in *A. thaliana* seedlings grown in Petri dishes, and also with the more recent measurements reported by Job et al. (2022). This trend of metabolite accumulation measured in Col-0 contrasted significantly with what reported in *B. rapa*, where almost all metabolites, with the only exception of the indolic glucosinolate 4-hydroxy-indolyl-3-methyl glucosinolate (4MOI3M), registered a temporal increase (**Fig. 6**; **Fig. S1**). Indolic glucosinolates, and in particular 1-methoxy-indolyl-3-methyl glucosinolate (1MOI3M), indolyl-3-methyl glucosinolate (I3M), and 4MOI3M, decreased also in *B. napus* upon UV-B treatment. As for the accumulation of anthocyanin derivatives, these compounds revealed a decreased accumulation upon UV-B treatment in all species, with the exception of *B. napus* (**Fig. S1**). We next focused attention on the accumulation of phenylacylated flavonoids, as the additional phenolic ring deriving from the sinapoyl decoration enhances the spectral absorption and consequently the UV-B shielding properties of glycosylated flavonoids (Tohge et al. 2016). In *A. thaliana*, all four classes of sinapoyl derivatives, which includes sinapoyl-glucoside (SinGlc), sinapoyl-malate (SinMal), di-sinapoyl-glucoside (Sin-Glc-Sin) and sinapoylated anthocyanin (A11, cyanidin 3-*O*-[2″-*O*-(2′″-*O*-(sinapoyl) xylosyl) 6″-*O*-(*p-O*-(glucosyl)-*p*-coumaroyl) glucoside] 5-*O*-[6″″-*O*-(malonyl) glucoside) revealed an increased accumulation with the exposure to UV-B (**Fig. 6**). In all the remaining Brassicaceae, only sinapoyl-glucoside derivatives revealed an increased accumulation in all species, while Sin-Glc-Sin and A11 were not detected.

In conclusion, all Brassicaceae species displayed the ability to mount a response to UV-B light, which includes transcriptional activation of the phenylpropanoid pathway, followed by the regulation of primary metabolism, ultimately culminating with synthesis and accumulation of specialized metabolites for the conferral of increased protection. As the analysis of primary metabolites revealed a widespread increase in Phe in all species, differences that we measured in the content of specific specialized metabolites could be attributed to species-specific activation of enzymes involved in the chemical decoration of flavonoid molecules.

## Conclusion

In this study, we examined the metabolic and transcriptional responses to UV-B radiation of four Brassicaceae species, including the model plant *A. thaliana* ecotype Col-0 and compared the results with responses in *B. napus*, *B. oleracea*, and *B. rapa*. Our analysis revealed a complete reprogramming of the pathway of the central metabolism, leading to a variation of metabolite abundance already visible after 2 h of UV-B treatment. Reprogramming of the central metabolism became even more evident after 10 h of exposure, revealing species-specific variation of metabolite abundance. Nonetheless, the immediate precursors of the flavonoid pathway and flavonoid decorations, such as the amino acid Phe, increased in all species. Species-specific variation was also observed in the content of specialized metabolites although sinapoyl glucose derivatives revealed a typical increase in all Brassicaceae species. Nonetheless, the analysis of *SCPL* transcript abundance displayed constitutive expression in all Brassicaceae and downregulation in response to the treatment with UV-B irradiation. Our study also suggested that the downregulation of *SCPL* expression is independent of the HY5 signaling cascade, which instead showed the conventional induction in all the Brassicaceae as previously reported for *A. thaliana*.

## Materials and Methods

### Plant material and growth

Seeds of *A. thaliana* ecotype Col-0, *B. napus*, *B. oleracea*, and *B. rapa* were surface-sterilized in 30% (v:v) bleach in water for 5 min, followed by a 5-min rinse with sterilized water. Sterilized seeds were sown on Petri dishes filled with half-strength Murashige and Skoog salts (Murashige and Skoog, 1962) containing 1% (w/v) sucrose and 1% agar (w/v) and vernalized at 4°C in the dark for 3 d. At the end of the vernalization period, the seeds were left to germinate for 10 d in a growth chamber set with the following conditions: light intensity, 180 μmol m^−2^s^−1^; photoperiod, 8/16 h:light/dark; temperature, 22°Cand humidity, 75%. Uniformly germinated seedlings were transferred to pots and grown under long-day conditions (photoperiod, 16/8 h:light/dark) for the full duration of the experiment. UV-B radiation (0.8 μmol m^−2^s^−1^; 315 nm) was applied to 2-week-old (transcriptional analysis) and 4-week-old (transcriptional and metabolomic analyses) seedlings starting at 9:00 AM when the plants had already received 3 h of visible photosynthetic radiation. Plant material was harvested at different time points as specified in the text using time 0 as a negative control. Measurements of metabolites were conducted in triplicate.

### Analysis of metabolites via GC-MS and LC-MS

Plant material was ground to fine powder using a ball mill (Tesch, Haan, Germany) at liquid N_2_ temperature and stored at −80°C until metabolite extraction. Primary and specialized metabolites were extracted from 50 mg of leaf powder with 0.7 ml of methanol containing ribitol (2 mg^.^l^−1^) and isovitexin (5 mg^.^l^−1^) as internal standards. A phase separation with chloroform was used to remove chlorophylls before separating each sample into two aliquots for the analysis of primary and specialized metabolites, respectively. Primary metabolites were derivatized with ethoxyamine hydrochloride in pyridine and measured via GC-MS following the procedure described by Lisec et al. (2006) and Alseekh et al. (2021). Specialized metabolites were separated and measured via LC-MS following the protocols previously described by Tohge and Fernie (2010) and Perez de Souza et al. (2021).

### Phylogenetic analysis of *SCPL* genes

Orthologs of *A. thaliana SCPL* genes in the Brassicaceae species were identified as previously described (Tohge et al. 2016). Sequences of *SCPL* genes were aligned with MUSCLE and used to build the phylogenetic tree in MEGA 7.0 using the maximum likelihood method. Short genes (CRU_004G02050, TP4G01870, TP4G01860, TP4G01990 and TP4G02020) were excluded from this analysis.

### Gene expression analysis by qRT-PCR

RNA extraction from plant tissues was performed using TRIzol reagent (ThermoFisher, Carlsbad, CA, USA). Digestion with DNase and cDNA synthesis were performed with Maxima First Strand cDNA Synthesis Kit with dsDNase (Thermo Fisher Scientific Baltics UAB, Vilnius, Lithuania). The integrity of the RNA was verified on 1% (w/v) agarose gels, and the concentration was measured using a Nanodrop ND-1000 spectrophotometer (Thermo Fisher Scientific, Wilmington, DE, USA). A final volume of 5 µl was used to run qRT-PCR reactions (Caldana et al. 2007). Data analysis was performed using SDS software version 2.4 (Applied Biosystem, Foster City, CA, USA) normalized to the expression of *UBQ10* housekeeping gene. The oligonucleotides used for the qRT-PCR analyses are provided in **Supplementary Table S3**. Three or six replicates were utilized to measure gene expression.

## Supplementary Material

pcad085_Supp

## Data Availability

Chromatograms will be made available upon direct request to the corresponding authors.

## References

[R1] Alber A.V., Renault H., Basilio‐Lopes A., Bassard J.E., Liu Z., Ullmann P., et al. (2019) Evolution of coumaroyl conjugate 3‐hydroxylases in land plants: lignin biosynthesis and defense. *Plant J.* 99: 924–936. 31038800 10.1111/tpj.14373

[R2] Alseekh S., Aharoni A., Brotman Y., Contrepois K., D’Auria J., Ewald J., et al. (2021) Mass spectrometry-based metabolomics: a guide for annotation, quantification and best reporting practices. *Nat. Methods* 18: 747–756. 34239102 10.1038/s41592-021-01197-1 PMC8592384

[R3] Caldana C., Scheible W.-R., Mueller-Roeber B. and Ruzicic S. (2007) A quantitative RT-PCR platform for high-throughput expression profiling of 2500 rice transcription factors. *Plant Methods* 3: 1–9. 17559651 10.1186/1746-4811-3-7 PMC1914063

[R4] Chanoca A., Kovinich N., Burkel B., Stecha S., Bohorquez-Restrepo A., Ueda T., et al. (2015) Anthocyanin vacuolar inclusions form by a microautophagy mechanism. *Plant Cell* 27: 2545–2559. 26342015 10.1105/tpc.15.00589 PMC4815043

[R5] Chapman J.M., Muhlemann J.K., Gayomba S.R. and Muday G.K. (2019) RBOH-dependent ROS synthesis and ROS scavenging by plant specialized metabolites to modulate plant development and stress responses. *Chem. Res. Toxicol.* 32: 370–396. 30781949 10.1021/acs.chemrestox.9b00028 PMC6857786

[R6] Dean J.C., Kusaka R., Walsh P.S., Allais F. and Zwier T.S. (2014) Plant sunscreens in the UV-B: ultraviolet spectroscopy of jet-cooled sinapoyl malate, sinapic acid, and sinapate ester derivatives. *J. Am. Chem. Soc.* 136: 14780–14795. 25295994 10.1021/ja5059026

[R7] Ghosh U.K., Islam M.N., Siddiqui M.N., Cao X. and Khan M.A. (2022) Proline, a multifaceted signalling molecule in plant responses to abiotic stress: understanding the physiological mechanisms. *Plant Biol.* 24: 227–239. 34796604 10.1111/plb.13363

[R8] Halliwell B. and Gutteridge J.M. (1985) Free Radicals in Biology and Medicine, 5th edn. Oxford University press, Pergamon.

[R9] Han X., Chang X., Zhang Z., Chen H., He H., Zhong B., et al. (2019) Origin and evolution of core components responsible for monitoring light environment changes during plant terrestrialization. *Mol Plant* 12: 847–862. 31009752 10.1016/j.molp.2019.04.006

[R10] Jenkins G.I. (2009) Signal transduction in responses to UV-B radiation. *Annu. Rev. Plant Biol.* 60: 407–431. 19400728 10.1146/annurev.arplant.59.032607.092953

[R11] Job N., Lingwan M., Masakapalli S.K. and Datta S. (2022) Transcription factors BBX11 and HY5 interdependently regulate the molecular and metabolic responses to UV-B. *Plant Physiol.* 189: 2467–2480. 35511140 10.1093/plphys/kiac195 PMC9342961

[R12] Kusano M., Tohge T., Fukushima A., Kobayashi M., Hayashi N., Otsuki H., et al. (2011) Metabolomics reveals comprehensive reprogramming involving two independent metabolic responses of Arabidopsis to UV-B light. *Plant Mol. Biol.* 67: 354–369. 10.1111/j.1365-313X.2011.04599.x 21466600

[R13] Lau K., Podolec R., Chappuis R., Ulm R. and Hothorn M. (2019) Plant photoreceptors and their signaling components compete for COP1 binding via VP peptide motifs. *EMBO J.* 38: e102140. 10.15252/embj.2019102140 PMC6745501 31304983

[R14] Lisec J., Schauer N., Kopka J., Willmitzer L. and Fernie A.R. (2006) Gas chromatography mass spectrometry–based metabolite profiling in plants. *Nat. Protoc.* 1: 387–396. 17406261 10.1038/nprot.2006.59

[R15] Muhlemann J.K., Younts T.L.B. and Muday G.K. (2018) Flavonols control pollen tube growth and integrity by regulating ROS homeostasis during high-temperature stress. *Proc. Natl. Acad. Sci. U.S.A.* 115: E11188–e11197. 30413622 10.1073/pnas.1811492115 PMC6255205

[R16] Murashige T. and Skoog F. (1962) A revised medium for rapid growth and bioassays with tobacco tissue cultures. *Physiol. Plant*. 15: 473–497.

[R17] Perez de Souza L., Alseekh S., Scossa F. and Fernie A.R. (2021) Ultra-high-performance liquid chromatography high-resolution mass spectrometry variants for metabolomics research. *Nat. Methods* 18: 733–746. 33972782 10.1038/s41592-021-01116-4

[R18] Podolec R., Demarsy E. and Ulm R. (2021) Perception and signaling of ultraviolet-B radiation in plants. *Annu. Rev. Plant Biol.* 72: 793–822. 33636992 10.1146/annurev-arplant-050718-095946

[R19] Podolec R., Wagnon T.B., Leonardelli M., Johansson H. and Ulm R. (2022) Arabidopsis B‐box transcription factors BBX20‐22 promote UVR8 photoreceptor‐mediated UV‐B responses. *Plant J.* 111: 422–439. 35555928 10.1111/tpj.15806 PMC9541035

[R20] Rai N., Neugart S., Yan Y., Wang F., Siipola S.M., Lindfors A.V., et al. (2019) How do cryptochromes and UVR8 interact in natural and simulated sunlight? *J. Exp. Bot.* 70: 4975–4990. 31100755 10.1093/jxb/erz236 PMC6760287

[R21] Rensing S.A. (2018) Great moments in evolution: the conquest of land by plants. *Curr. Opin. Plant Biol.* 42: 49–54. 29525128 10.1016/j.pbi.2018.02.006

[R22] Rizzini L., Favory J.J., Cloix C., Faggionato D., O’Hara A., Kaiserli E., et al. (2011) Perception of UV-B by the Arabidopsis UVR8 protein. *Science* 332: 103–106. 21454788 10.1126/science.1200660

[R23] Ruegger M. and Chapple C. (2001) Mutations that reduce sinapoylmalate accumulation in *Arabidopsis thaliana* define loci with diverse roles in phenylpropanoid metabolism. *Genetics* 159: 1741–1749. 11779811 10.1093/genetics/159.4.1741 PMC1461910

[R24] Sakamoto S., Kamimura N., Tokue Y., Nakata M.T., Yamamoto M., Hu S., et al. (2020) Identification of enzymatic genes with the potential to reduce biomass recalcitrance through lignin manipulation in Arabidopsis. *Biotechnol. Biofuels* 13: 1–6. 32514309 10.1186/s13068-020-01736-6 PMC7260809

[R25] Shi C. and Liu H. (2021) How plants protect themselves from ultraviolet-B radiation stress. *Plant Physiol.* 187: 1096–1103. 34734275 10.1093/plphys/kiab245 PMC8566272

[R26] Stracke R., Favory J.J., Gruber H., Bartelniewoehner L., Bartels S., Binkert M., et al. (2010) The Arabidopsis bZIP transcription factor HY5 regulates expression of the PFG1/MYB12 gene in response to light and ultraviolet-B radiation. *Plant Cell Environ.* 33: 88–103. 19895401 10.1111/j.1365-3040.2009.02061.x

[R27] Tohge T. and Fernie A.R. (2010) Combining genetic diversity, informatics and metabolomics to facilitate annotation of plant gene function. *Nat. Protoc.* 5: 1210–1227. 20539294 10.1038/nprot.2010.82

[R28] Tohge T. and Fernie A.R. (2017) Leveraging natural variance towards enhanced understanding of phytochemical sunscreens. *Trends Plant Sci.* 22: 308–315. 28173981 10.1016/j.tplants.2017.01.003

[R29] Tohge T., Perez de Souza L. and Fernie A.R. (2018) On the natural diversity of phenylacylated-flavonoid and their in planta function under conditions of stress. *Phytochem. Rev.* 17: 279–290. 29755304 10.1007/s11101-017-9531-3 PMC5932100

[R30] Tohge T., Wendenburg R., Ishihara H., Nakabayashi R., Watanabe M., Sulpice R., et al. (2016) Characterization of a recently evolved flavonol-phenylacyltransferase gene provides signatures of natural light selection in Brassicaceae. *Nat. Commun.* 7: 12399. 10.1038/ncomms12399 PMC4996938 27545969

[R31] Ulm R., Baumann A., Oravecz A., Máté Z., Adám E., Oakeley E.J., et al. (2004) Genome-wide analysis of gene expression reveals function of the bZIP transcription factor HY5 in the UV-B response of Arabidopsis. *Proc. Natl. Acad. Sci. U.S.A.* 101: 1397–1402. 14739338 10.1073/pnas.0308044100 PMC337064

[R32] Waszczak C., Carmody M. and Kangasjärvi J. (2018) Reactive oxygen species in plant signaling. *Annu. Rev. Plant Biol.* 69: 209–236. 29489394 10.1146/annurev-arplant-042817-040322

[R33] Yin R., Arongaus A.B., Binkert M. and Ulm R. (2015) Two distinct domains of the UVR8 photoreceptor interact with COP1 to initiate UV-B signaling in Arabidopsis. *Plant Cell* 27: 202–213. 25627067 10.1105/tpc.114.133868 PMC4330580

