# Supplementary material for: Cross-Species Metabolomic Analyses in the Brassicaceae Reveals Common Responses to Ultraviolet-B Exposure
Source: Plant Cell Physiol. 2023 Aug 12;64(12):1523–33. doi: 10.1093/pcp/pcad085 (PMC10734891; doi:10.1093/pcp/pcad085)
Supplement: pcad085_Supp [file pcad085_supp.zip › suppl_data/pcp-2023-e-00025-File008.pdf]

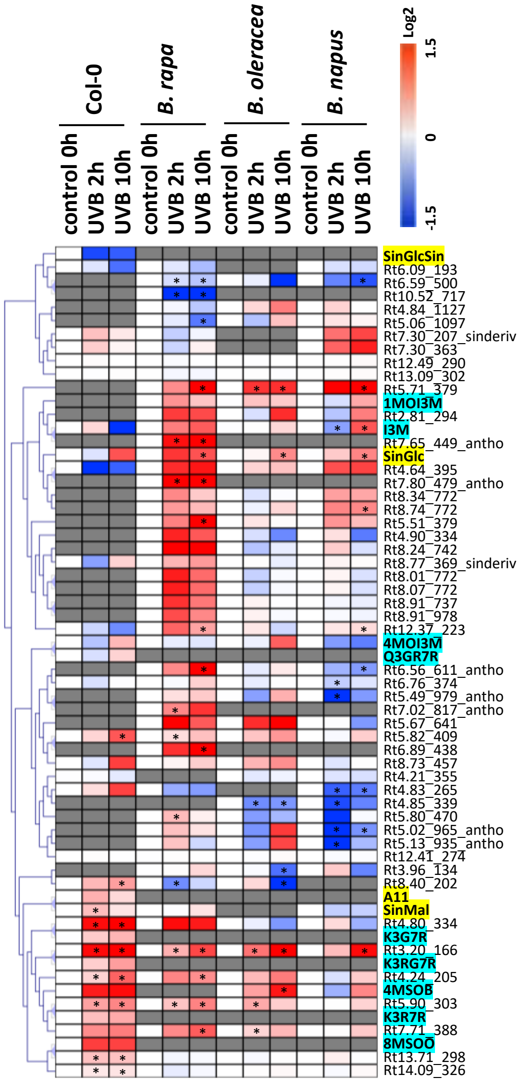

Figure S1. Heatmap of metabolite abundance measured in leaves of *A. thaliana* ecotype Col-0, *B. napus*, *B. oleracea* and *B. rapa* exposed to UV-B radiation. Each square in the heatmap represent log_2 values of metabolite abundance. Data are represented as fold change of metabolite abundance versus time 0. Metabolites marked with an asterisk are significantly different at $P < 0.05$. Abbreviations: A11, cyanidin 3-*O*-[2''-*O*-(2'''-*O*-(sinapoyl) xylosyl) 6''-*O*-(*p*-*O*-(glucosyl)-p-coumaroyl) glucoside] 5-*O*-[6''''-*O*-(malonyl) glucoside; G, glocoside; I3M, indolyl-3-methyl glucosinolate; K, kaempferol; Mal, malate; Q, quercetin; R, rhamnoside; Sin, sinapoyl; 1MOI3M, 1-methoxy-indolyl-3-methyl glucosinolate; 4MOI3M, 4-hydroxy-indolyl-3-methyl glucosinolate; 4MSOB, 4-methylsulfinylbutyl glucosinolate; 8MSOO, 8-methylsulfinyloctyl glucosinolate. Metabolites highlighted in blue and yellow are represented as bar plots in Fig. 6.
